# A cross-tissue splicing signature as a quantitative biomarker for ReNU syndrome

**DOI:** 10.64898/2026.09.26.26364077

**Published:** 2026-09-29

**Authors:** Ruebena Dawes, Alexander Blakes, Theodora Markati, Michael Griffiths, Benjamin Cogné, Tahsin Stefan Barakat, Jeffrey C Barrett, Carlo Rinaldi, Stephan J Sanders, Nicola Whiffin

## Abstract

ReNU syndrome is a severe neurodevelopmental disorder caused by *de novo* variants in the spliceosomal small nuclear RNA (snRNA) gene *RNU4-2.* Pathogenic variants cluster in two distinct structural regions of the U4 snRNA produced by *RNU4-2* , the T-loop and Stem III. Aberrant 5’ splice site selection is a molecular hallmark of ReNU syndrome that correlates with phenotypic severity and could be leveraged as a quantitative biomarker to facilitate preclinical and clinical therapeutic development. We analysed RNA-sequencing data from two independent cohorts totalling 30 individuals with ReNU syndrome and 54 controls. We identified 483 alternative splicing events shared across both cohorts, with the highest concordance in 105 alternative 5’ splice-site (A5SS) events. Using these 105 A5SS events, we derived a minimal reproducible signature from only six sites. The splicing signature perfectly distinguished 19 n.64_65insT individuals from 54 controls, and produced no false positives when applied to a further 5,984 whole blood controls. In addition, the signature was specific to variants within the U4-2 T-loop, which are associated with greater clinical severity. Further, we find evidence of alternative splicing events that are specific to variants in Stem III. In a patient-derived induced pluripotent stem cell (iPSC) model of the recurrent *RNU4-2* n.64_64insT variant, the splicing signature was fully recapitulated across all stages of differentiation into cortical neuron-like cells. Together, these findings define a robust RNA biomarker for T-loop ReNU syndrome for use in therapeutic development, and illustrate a general framework for portable biomarker discovery in spliceopathies.

## Full Text Availability

The license terms selected by the author(s) for this preprint version do not permit archiving in PMC. The full text is available from the preprint server.

